# Transposon Assisted Gene Insertion Technology (TAGIT): A Tool for Generating Fluorescent Fusion Proteins

**DOI:** 10.1371/journal.pone.0008731

**Published:** 2010-01-15

**Authors:** James A. Gregory, Eric C. Becker, James Jung, Ida Tuwatananurak, Kit Pogliano

**Affiliations:** Division of Biological Sciences, University of California San Diego, La Jolla, California, United States of America; University of Massachusetts, United States of America

## Abstract

We constructed a transposon (transposon assisted gene insertion technology, or TAGIT) that allows the random insertion of *gfp* (or other genes) into chromosomal loci without disrupting operon structure or regulation. TAGIT is a modified Tn5 transposon that uses Kan^R^ to select for insertions on the chromosome or plasmid, β-galactosidase to identify in-frame gene fusions, and Cre recombinase to excise the *kan* and *lacZ* genes *in vivo*. The resulting *gfp* insertions maintain target gene reading frame (to the 5′ and 3′ of *gfp*) and are integrated at the native chromosomal locus, thereby maintaining native expression signals. Libraries can be screened to identify GFP insertions that maintain target protein function at native expression levels, allowing more trustworthy localization studies. We here use TAGIT to generate a library of GFP insertions in the *Escherichia coli* lactose repressor (LacI). We identified fully functional GFP insertions and partially functional insertions that bind DNA but fail to repress the *lacZ* operon. Several of these latter GFP insertions localize to *lacO* arrays integrated in the *E. coli* chromosome without producing the elongated cells frequently observed when functional LacI-GFP fusions are used in chromosome tagging experiments. TAGIT thereby faciliates the isolation of fully functional insertions of fluorescent proteins into target proteins expressed from the native chromosomal locus as well as potentially useful partially functional proteins.

## Introduction

Recent advances in optical microscopy enable fluorescently tagged proteins to be observed with subdiffraction-limited spatial resolution and outstanding temporal resolution. The combination of Photo Activated Localization Microscopy (PALM) and Stochastic Optical Reconstruction Microscopy (STORM) provides a ten-fold gain in spatial resolution and allows individual proteins to be counted [1]–[5]. However, achieving the maximum gain from these methods requires that the behavior of the fluorescently-tagged fusion protein accurately represents that of the native protein.

Studies of protein localization in living cells are often compromised by protein overproduction or by partially functional fusion proteins (reviewed by [6], [7]). Examples of partially functional fusion proteins include GFP fusions to the *B. subtilis* engulfment proteins, which cause synergistic engulfment defects [8] and GFP fusions to FtsZ, which are temperature sensitive in most species, including *B. subtilis* [9]. Co-expressing tagged and untagged proteins is a frequently-used solution that makes it impossible to use PALM/STORM techniques to quantify the number of molecules at a particular location, since the complex will be a mixture of untagged and tagged protein. Overexpression can also cause misleading protein localization. A two-fold overexpression of a partially functional GFP-SpoIIQ fusion protein changes its localization [10]. Overexpression of *Bacillus subtilis* MinC causes it to accumulate at the cell poles [11], [12], although when produced under its native expression controls MinC localizes to midcell [13]. Furthermore, even modest overproduction of some proteins, particularly those involved in signal transduction and cell division, can have deleterious effects on cell viability and on cellular architecture.

The ideal strategy for imaging studies is to employ fully functional fluorescent fusion proteins produced from a gene in its native chromosomal context. This is difficult to achieve using existing technologies, which typically use conventional molecular biology techniques to fuse *gfp* to the 5′ or 3′ end of the target gene [14]–[16]. It is particularly difficult to maintain appropriate expression of genes encoded in bacterial operons, which can be transcribed from several promoters and in which translation of consecutive genes can depend on overlapping translation signals.

**Figure 1:**
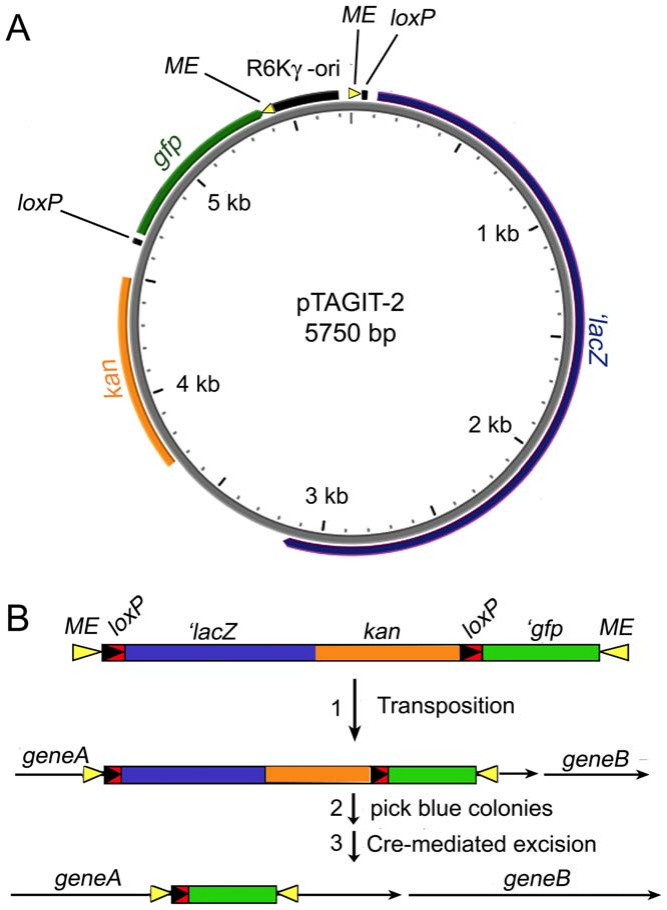
Structure of TAGIT, which randomly inserts *gfp* into target genes. (A) pTAGIT-2 is an independently replicating plasmid that was constructed by ligating TAGIT-2 to the R6Kγ origin of replication. (B) ME = mosaic ends recognized by Tn5 transposase (yellow arrowheads), *loxP* = recognition sequences for Cre recombinase (black arrowheads on red), *kan* = encodes Kan^R^ in *B. subtilis* and *E. coli* (orange). *gfp* = gene for GFP mutant 2 (green). *'lacZ* = gene for β-galactosidase lacking the translational initiation codon (blue). GFP is not fluorescent when exported via the Sec pathway, so the use of β-galactosidase, which is also only active in the cytoplasm, allows the isolation of GFP insertions into the cytoplasmic domain of membrane proteins. A single ORF extends through the leftward ME and *loxP* site into *'lacZ*, so that after transposition, β-galactosidase will be expressed only if TAGIT has inserted in the correct reading frame. Both *loxP* sites are in the same reading frame, so that after excision of the *'lacZ* and *kan* genes by the Cre recombinase, *gfp* is in the same reading frame as was *lacZ* so that translation can continue out of *gfp* and into the 3′ end of the target gene. The resulting proteins have GFP inserted into the middle of the target protein.

One approach to solving this problem is to randomly insert GFP into target genes and then screen for GFP insertions that maintain target protein function [17]–[22]. We developed a variation on this approach that allows the random insertion of *gfp* into target genes in their normal chromosomal context, without disrupting expression of upstream or downstream genes. This method, which we call TAGIT (transposon assisted gene insertion technology), allows rapid isolation of in-frame hybrid genes (Figure 1). The resulting genes encode “sandwich” fusion proteins in which GFP is inserted into the middle of a protein; we call these fusions “GFP insertions” (abbreviated GFPi), to distinguish them from N- or C-terminal GFP fusions. The feasibility of sandwich fusions was originally demonstrated for MalF, an integral membrane protein component of the maltose-maltodextrin transport system [23]. Insertion of alkaline phosphatase into MalF produced a hybrid protein retaining both alkaline phosphatase and maltose transport activities. GFP is well-suited for the construction of sandwich fusions, because its N- and C-termini are close to one another [24]. We built TAGIT to take advantage of this feature and to facilitate the construction of GFP sandwich fusions expressed from the native chromosomal locus to avoid protein overproduction artifacts.

TAGIT offers several advantages to previously published GFP transposons [17]–[22], [25]. First, TAGIT includes the *lacZ* gene to allow the rapid identification of in-frame insertions and significantly reduce the number of insertions screened. Second, TAGIT allows removal of the selectable marker necessary to isolate transposition events and *lacZ* using the Cre recombinase [26] rather than restriction enzymes. Cre is functional in bacterial and eukaryotic cells and therefore allows excision of selectable markers on the chromosome of living cells. Thus, TAGIT generates fluorescent insertion genes that maintain their native expression signals rather than utilizing inducible promoters. Together these modifications eliminate the time and resource intensive processes of identifying in-frame fusions with DNA sequencing and excising selectable markers *in vitro* using restriction endonucleases, which hinders future efforts to integrate the fusions into chromosomal loci.

We here demonstrate that TAGIT can be used to isolate internal insertions of GFP into a target protein, using the *Escherichia coli* lactose repressor (LacI) as a test case. LacI is an ideal candidate because of extensive studies of its function, structure, and regulation [27]–[29]. Furthermore, previous epitope insertion mutagenesis of *lacI* identified linker regions within LacI capable of tolerating a 31 amino acid insertion [30], which we reasoned might also tolerate GFP insertion. After constructing a library of LacI-GFP insertion proteins (LacI-GFPi) using TAGIT, we identified six sites in LacI that are tolerant to GFP insertion, including those previously identified by epitope insertion mutagenesis. We also isolated several insertions that maintained the ability to bind to the *lac* operator, but were unable to repress the *lac* operon. These partially functional LacI-GFPi proteins could potentially be used to track chromosome dynamics without the affects on chromosome segregation sometimes observed for fully functional LacI-GFP fusions [31].

## Results

### Construction of TAGIT

TAGIT consists of five elements that together allow identification of in-frame insertions and the subsequent *in vivo* removal of marker genes to construct a library of *gfp* insertions within a target gene (Figure 1). (1) At either end of TAGIT are the optimized minimal inverted repeats (19 bp mosaic ends; ME) that allow the hyperactive Tn5 transposase to mediate transposition [32]. (2) Near the 5′ end of TAGIT is the *'lacZ* gene, which lacks translational initiation sequences, such that β-galactosidase is only expressed after insertion into an open reading frame. (3) Encoded downstream of *'lacZ* is an aminoglycoside phosphotransferase (*kan*) gene, which confers resistance to kanamycin (Kan^R^) in both *B. subtilis* and *E. coli*, allowing selection for transpositions in either organism. (4) Near the 3′ end of TAGIT is the *gfp* gene, which also lacks translational initiation sequences. (5) Finally, two *loxP* sites are within the transposon, the first immediately upstream of *'lacZ* and the second immediately downstream of *kan* and upstream of *gfp*. These *loxP* sites allow Cre recombinase [33] to mediate excision of *'lacZ* and *kan* either *in vivo* or *in vitro*. The delivery vector for TAGIT, pTAGIT-1, contains the R6Kγ origin of replication, which functions only in *E. coli* strains expressing the *pir* gene [13], [34].

TAGIT-1 (the first version of TAGIT) contains a single open reading frame extending through the leftward ME and *loxP* site into *'lacZ*. This ensures that β-galactosidase will be expressed only if TAGIT has inserted into an expressed open reading frame. In addition, the rightward *loxP* site is in the same reading frame as the leftward *loxP* site, and this reading frame continues through *gfp* and the rightward ME, into the target gene. Thus, after Cre-mediated excision, the *gfp* gene maintains the same reading frame as the excised *lacZ* gene, and translation continues out of *gfp* and into the 3′ end of the target gene. The resulting genes therefore encode ‘sandwich’ fusion proteins; we call these fusions “GFP insertions”, to distinguish them from conventional N- or C-terminal fusions.

### Isolation of GFP Insertions in LacI

We have used TAGIT-1 to construct *gfp* insertions into the *B. subtilis minCD* operon [13], but when we attempted to use TAGIT-1 to isolate in-frame insertions on *E. coli* plasmids, we found that both in-frame and out-of-frame TAGIT insertions produced indistinguishable levels of β-galactosidase activity. We solved this issue by first mutagenizing pTAGIT-1 to change an internal ATG codon at codon 3 of *lacZ*, which provides a potential internal translational initiation codon to GCG (which encodes alanine) and by lowering the copy number of the plasmid by using an *E. coli pcnB* strain [35]. We named the resulting transposon TAGIT-2.

**Figure 2:**
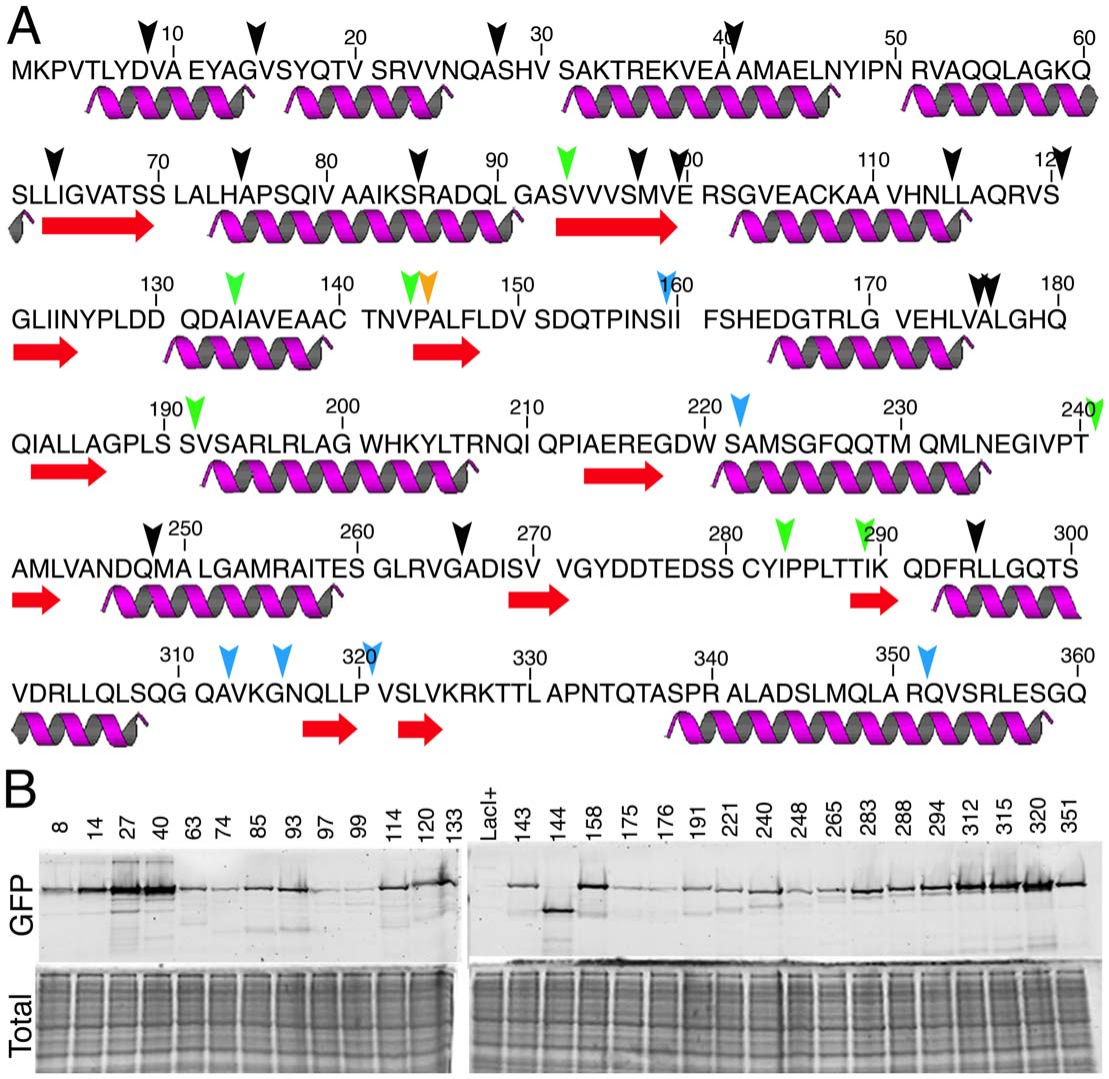
Analysis of TAGIT-2-constructed GFP insertions in the *E. coli* lactose repressor LacI. (A) Amino acid sequence and structural features of LacI, with purple helices indicating α−helices and red arrows indicating β-sheets. Black arrowheads indicate the position of non-functional GFP insertions (Repression−, Focus−). Green arrowheads indicate GFP insertions that fail to repress *lacZ*, but form foci (Repression−, Focus+) when introduced into a strain with the *lacO* array integrated near the terminus of replication. Blue arrowheads indicate insertions that repress *lacZ* (Repression+, Focus+). The orange arrowhead indicates the position of the GFP insertion that is out of frame on the 3′ side of GFP. (B) The LacI-GFP insertions accumulate to variable levels. Numbers correspond to the last undisrupted *lacI* codon before TAGIT. Cells were harvested at an OD_600_ of ∼0.5, samples prepared and subject to SDS-PAGE. Protein accumulation was determined using in-gel GFP fluorescence (top panel) and the gel was subsequently stained with Coomassie blue to reveal total protein (bottom panel).

We performed *in vitro* transposition with pTAGIT-2 into the *lacI^q^* containing plasmids pEB363 and pEB364 (which have *lacI* inserted in opposite orientations relative to the plasmid backbone) using purified Tn5 transposase [32]. The resulting insertion library was transformed into the *pcnB* strain KJ622, selecting for Kan^R^ on plates containing the β-galactosidase indicator X-gal. DNA sequencing revealed that 100% of the 57 blue colonies purified for sequencing contained insertions in the same reading frame as the target gene, *lacI*. The 30 unique insertion sites were distributed throughout *lacI* and provided sufficient coverage of LacI (Figure 2) to compare with previously constructed epitope insertion mutants of LacI [30]. One of these *gfp* insertions (LacI-144-GFP) was found to be out of frame on the 3′ side of *gfp*, thereby producing a protein containing the first 144 amino acids of LacI followed by GFP, because the frame shift resulted in a stop codon following the 3′ end of *gfp*. Thus, the incorporation of *'lacZ* into TAGIT allows the rapid and accurate identification of in-frame *gfp* insertions prior to sequencing, thereby reducing the cost and effort required to identify in-frame insertions.

We excised *kan* and *'lacZ in vivo* with Cre recombinase [26] by transforming the plasmids into an *E. coli* strain that transiently expresses Cre (Materials and Methods). The transformants were selected on ampicillin (*bla* is encoded on the plasmid backbone) and tested for kanamycin sensitivity to ensure that excision had occurred. Successful excision occurred in approximately 80% of transformants (data not shown). We used Cre for excision of *kan* and *'lacZ* because the 21 base pair *loxP* sites are unlikely to be present in the target gene. It also eliminates the time consuming process of isolating plasmid DNA, performing a restriction digest, and transforming *E. coli* with the ligated plasmid. Finally, restriction enzymes must be used *in vitro* on plasmid DNA and ultimately results in the loss of the selectable marker, thus eliminating the possibility of integrating the gene into the native chromosomal locus. Cre can be expressed *in vivo* after integration of the modified gene into the native locus, thereby leaving the chromosomal structure intact and maintaining the native expression signals.

### Test of the LacI-GFP Insertion Proteins for Function

We first tested if the LacI-GFP insertion proteins were able to repress the *lacZYA* operon in *E. coli* strain CSH140, in which a *lacI* mutation renders expression constitutive [36]. We transformed CSH140 with the control plasmids pEB363 or pEB364 (in opposite orientations) and the TAGIT-2 constructed GFP insertions and performed β-galactosidase assays on strains cultured in the absence of lactose or IPTG. Repression activity was calculated as the ratio of the β-galactosidase activity (Miller units) produced by the parent strain (CSH140) to that of the transformant. LacI produced from pEB363 and pEB364 repressed the *lac* operon with equivalent efficiency and the combined data is shown in Table 1 for the LacI+ control. Six of the thirty unique LacI-GFPi proteins repressed the *lac* operon at least two-fold over background (GFP insertions at amino acid 158, 221, 312, 315, 320, and 351), with the most active, LacI-312-GFPi, showing a repression activity of 3,100. Four of the six of repression-competent GFP insertions were also induced by IPTG, increasing β-galactosidase activity 2–8 fold. Two repression competent insertions, LacI-158-GFPi and LacI-221-GFPi, were not induced by IPTG, suggesting that the GFP insertion interferes with inducer binding [27]. We conclude that six of our GFP insertions retained a significant amount of repression activity and that most of these are inducible.

**Table 1:** *In vivo* activities and accumulation of LacI-GFP insertion proteins.

Insertion site	Repression Activity^1^	Inducibility^2^	Relative Protein Level^3^	Localization
LacI+	16,500±5800	74.4±7.1	NA^4^	NA
LacI8-GFPi	0.814±0.03	NA	6.84	Cytoplasmic
LacI14-GFPi	0.921±0.09	NA	14.2	Cytoplasmic
LacI27-GFPi	0.867±0.04	NA	23.0	Inclusion bodies
LacI40-GFPi	0.916±0.10	NA	20.5	Inclusion bodies
LacI63-GFPi	1.03±0.12	NA	5.19	Inclusion bodies
LacI74-GFPi	0.812±0.05	NA	3.04	Cytoplasmic
LacI85-GFPi	1.27±0.64	NA	5.83	Inclusion bodies
LacI93-GFPi	0.908±0.16	NA	8.97	DNA foci
LacI97-GFPi	0.928±0.09	NA	1.83	Cytoplasmic
LacI99-GFPi	0.979±0.12	NA	1.00	Cytoplasmic
LacI114-GFPi	0.924±0.12	NA	7.68	Cytoplasmic
LacI120-GFPi	0.890±0.10	NA	8.50	Cytoplasmic
LacI133-GFPi	1.19±0.42	NA	12.1	DNA foci
LacI143-GFPi	1.10±0.13	NA	5.34	DNA foci
LacI144-GFPi	1.14±0.31	NA	10.9	Cytoplasmic
LacI158-GFPi	2.31±0.42	0.863±0.05	11.7	DNA foci
LacI175-GFPi	0.978±0.07	NA	2.01	Cytoplasmic
LacI176-GFPi	1.02±0.08	NA	1.47	Cytoplasmic
LacI191-GFPi	0.984±0.12	NA	3.49	DNA foci
LacI221-GFPi	7.95±0.54	0.789±0.48	3.42	DNA foci
LacI240-GFPi	1.14±0.10	NA	6.33	DNA foci
LacI248-GFPi	1.23±0.04	NA	3.34	Cytoplasmic
LacI265-GFPi	1.27±0.10	NA	4.29	Cytoplasmic
LacI283-GFPi	1.27±0.09	NA	9.91	DNA foci, inclusion bodies
LacI288-GFPi	1.14±0.05	NA	7.75	DNA foci, inclusion bodies
LacI294-GFPi	1.32±0.08	NA	11.1	Inclusion bodies
LacI312-GFPi	3110±730	2.09±0.33	16.3	DNA foci
LacI315-GFPi	1660±98	2.59±0.32	16.7	DNA foci
LacI320-GFPi	9.09±0.39	7.38±1.7	19.6	DNA foci
LacI351-GFPi	2000±160	3.37±0.33	11.1	DNA foci

1 Repression activity = β-gal activity of CSH140 divided that of the indicated plasmid in CSH140. Cells were grown in the absence of IPTG. At least three cultures were assayed.

2 Inducibility = Repression activity of cells grown in the absence of IPTG divided by that of cells grown in the presence of IPTG. At least three cultures were assayed.

3 Protein levels were determined by quantifying the gel shown in Figure 2B. Relative protein levels were calculated by dividing each sample by the protein level of LacI-99-GFPi, which had the lowest protein accumulation.

4 NA = Not applicable.

### Relative Protein Abundance of LacI-GFP Insertion Proteins

The insertion of GFP into domains of LacI critical for folding might decrease the stability and accumulation of the LacI-GFPi proteins thereby decreasing repression activity. To determine if variations in the accumulation of the GFP insertion proteins was responsible for the variations in repression activity, we quantified the amount of protein being produced by each *lacI-gfp* insertion. Protein samples were prepared from each strain for in-gel detection of GFP [37] and accumulation was quantified using a Typhoon 9400 (Materials and Methods). We observed one major band of ∼70 kD in all samples except the frame-shift mutant LacI-144-GFP (which migrated at ∼50 kD). There was little variability in the apparent size of LacI-GFPi proteins and little evidence of degradation products (Figure 2B). The amount of protein varied approximately twenty-fold across all the samples, but protein levels did not correlate with repression activity (Table 1). For example, the highest and lowest protein levels (LacI-27-GFPi and LacI-99-GFPi respectively) were observed for nonfunctional proteins, while the repression activity of LacI-320-GFPi was decreased by nearly 300-fold compared to LacI-312-GFPi, although it accumulated at higher levels. This suggests that variations in the repression activity of the GFP insertion proteins is due to the position at which GFP is inserted, not to the level at which the protein accumulates.

### Localization of LacI-GFP Insertion Proteins

**Figure 3:**
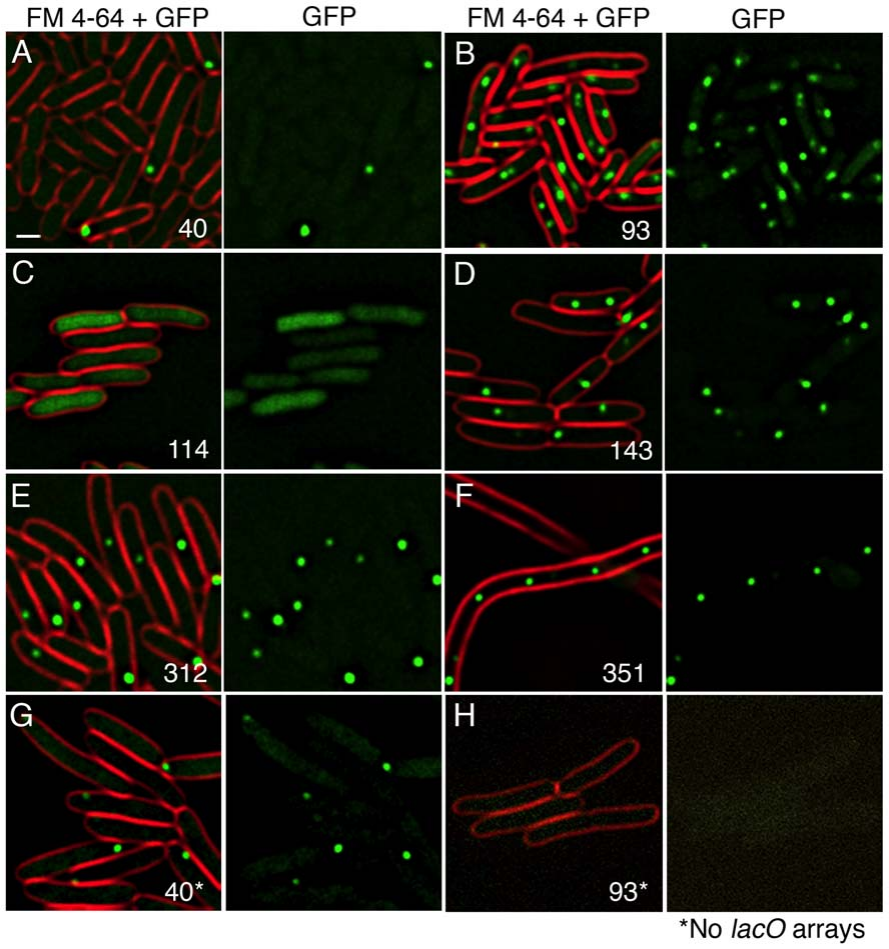
Binding of LacI-GFPi proteins to *lacO* arrays near the *E. coli* terminus of replication in growing cells. Numbers correspond to the location of GFP insertion into LacI. (A) LacI-40-GFPi localizes in foci near the cell poles, typical of inclusion bodies. (B) LacI-93-GFPi localizes as foci at midcell. (C) LacI-114-GFPi localizes to the cytoplasm. (D) LacI-143-GFPi, (E) LacI-312-GFPi localize as foci at midcell. (F) LacI-351-GFPi localizes as foci in filamentous cells. (G) LacI-40-GFPi forms foci in the absence of the *lacO* arrays (in strain CSH140), while (H) LacI-93-GFPi no longer assembles foci. Scale bar in A, 1 µm.

We next tested the ability of the LacI-GFPi proteins to bind the *lac* operator (*lacO*) in living cells. We introduced TAGIT-2 derived *lacI-gfp* insertion alleles into an *E. coli* strain that contains tandem copies of *lacO* integrated near the chromosomal terminus of replication (*ter*) [31]. LacI-GFPi proteins that are capable of binding *lacO* should assemble discrete foci near *ter*, which is located near midcell for most of the cell cycle [31]. Fluorescence microscopy demonstrated three classes of localization (Figure 3, Figure S1 shows localization of all LacI-GFPi proteins). One class showed irregularly sized foci that were typically localized near each cell pole or randomly positioned within the cell (Figure 3A, Figure S1). These are likely to be inclusion bodies, which accumulate near the cell poles [38], [39], because the formation of foci did not depend on the presence of *lacO* arrays (Figure 3G). Some GFP insertions appeared to contain both inclusion bodies and DNA bound foci, perhaps because the proteins did not fold efficiently and aggregate to form inclusion bodies (Figure S1, insertions 283 and 285). A second class showed cytoplasmic fluorescence, which likely indicates that the proteins failed to bind *lacO* (Figure 3C, Figure S1). A third class showed fluorescent foci that localized to midcell and were regularly spaced within the cells, as would be expected for proteins that bound the *ter*-proximal *lacO* array (Figure 3B,D–F, Figure S1). This class included all of the LacI-GFPi proteins that repressed the lactose operon. Surprisingly it also included several fusions that failed to repress the lactose operon, including GFP insertions at amino acid 93, 133, 143, 191, and 240 (Figure 3B, Table 1, Figure S1), suggesting that these proteins were able to bind *lacO*, but could not mediate repression. As expected, focus formation for these insertions depended on the presence of the *lacO* array (Figure 3H). We conclude that many GFP insertions in LacI maintain both GFP fluorescence and the ability of LacI to bind *lacO* DNA.

### LacI-GFPi Proteins That Disrupt Cell Division

Studies of chromosome and plasmid dynamics during cell growth have become increasingly dependent on the ability to track movement of DNA by fusing GFP to DNA binding proteins that recognize specific DNA sequences. LacI-GFP has been used extensively for such studies, but it can cause defects in cell division when a *lacO* array is integrated into the chromosome [31]. Indeed, we noted that many of the repression competent insertions showed various degrees of filamentation during growth (Figure 3F; Figure S1). Increasing growth temperature generally exacerbated this phenotype. However, several of our newly isolated GFP insertions alleviated the filamentation associated with *lacO* arrays and localized to DNA associated foci, including insertions at amino acids 93, 133, 143, 191, 221, 240 (Figure 3B, 3D, Figure S1). These GFP insertions could provide ideal tools for non-disruptive DNA tagging experiments.

## Discussion

We successfully used TAGIT to randomly generate *gfp* insertions into the *E. coli* lactose repressor (LacI) and identified LacI-GFPi proteins that maintain GFP fluorescence and various levels of LacI repressor activity. The incorporation of the *'lacZ* gene into TAGIT facilitated the rapid identification of 57 in-frame *gfp* insertions into *lacI*, which represented 30 unique insertion sites across *lacI*. The effect of GFP insertion on LacI activity was largely consistent with genetic and structural information available for LacI [27]–[29]. The most active LacI-GFPi protein contained GFP inserted after amino acid 312, 48 amino acids before the end of the protein. Therefore LacI activity was best preserved when GFP was inserted within the protein. We also isolated LacI-GFPi proteins that lost the ability to repress the lactose operon, but retained the ability to bind a *lacO* array integrated into the *E. coli* chromosome. These proteins alleviate the filamentation associated with more active LacI-GFP fusions and therefore could provide a less disruptive method to track movement of chromosome loci. Thus, TAGIT is a useful molecular tool that can be used to rapidly generate a library of GFP insertion proteins, which can subsequently be screened to isolate fully functional GFP insertion proteins as well as mutant proteins with novel biological activities.

### Analysis of LacI-GFPi Proteins

**Figure 4:**
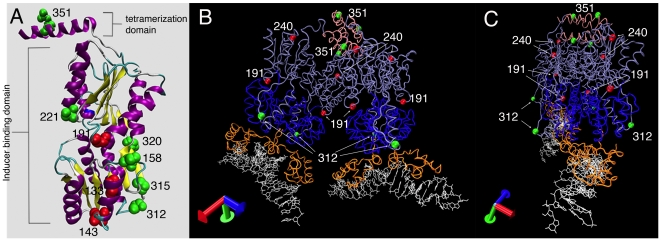
Cartoon of LacI-GFPi proteins mapped onto the crystal structure of LacI. (A) The monomeric structure of LacI without the DNA binding domain (PDB ID: 1LBI). All LacI-GFPi proteins that localize as foci were mapped onto a ribbon representation of the LacI crystal structure. The amino acid corresponding to the site of GFP insertion is labeled and represented as a space-filling model. Red amino acids correspond to insertion proteins that are unable to repress the *lac* operon and green amino acids correspond to insertion proteins that retain some level of repression activity. (B) Model of the LacI crystal (PDB ID: 1LBG) structure including the DNA (white), DNA binding domain (orange), N-terminal core domain (blue), C-terminal core domain (light purple), and tetramerization domain (pink). The most active GFP insertion (LacI-312-GFPi) and LacI-351-GFPi are shown as green balls and two examples of inactive GFP insertions as red balls. (C) Same as in (B) but rotated to show the projection of amino acid 312 from the surface.

We characterized thirty unique GFP insertions in the lactose repressor. Not surprisingly, most GFP insertions produced nonfunctional proteins. However, several retained LacI repressor activity while others bound DNA but failed to repress the *lac* operon. In the next section, we analyze the insertions with respect to the domain structure of the LacI protein (Figure 4) [27]–[29], [40].

#### DNA binding domain

The N-terminus of LacI consists of four helices that together comprise the DNA binding domain (residues 1–62; not shown in Figure 4) of LacI and a linker to the core domain [40]. This region has been previously identified as sensitive to mutation and substitution. We therefore expected this region to be intolerant of GFP insertion. Indeed, the four insertions we isolated in this domain (GFP insertions at amino acid 8, 14, 27, and 40) showed no repression activity and localized to the cytoplasm or inclusion bodies. LacI-27-GFPi and LacI-40-GFPi had the highest relative protein accumulation, which could account for the presence of inclusion bodies.

#### Inducer binding domain

The inducer-binding domain of LacI contains two separate subdomains of similar structure, the N-terminal core domain (residues 61–163 and 293–320) and the C-terminal subdomain (residues 164–292). Both domains contribute to a six stranded parallel β–sheet located between four alpha helices [29]. The N-terminal core domain contains four regions that are highly tolerant to substitutions (amino acids 100–112, 129–145, 151–160, and 305–318 [41]) and to epitope insertions at amino acids152 and 317 [30]. We therefore predicted that these regions were likely to tolerate GFP insertion. Indeed, LacI-312-GFPi (Figure 4B and 4C) is the most active repressor that we isolated from our screen and the repression activity of LacI-315-GFPi is just two-fold lower than LacI-312-GFPi. Interestingly, LacI-320-GFPi is approximately 300 fold less active than LacI-312-GFPi, which correlates well with a decreased tolerance for substitution from residues 319–330 [27]. LacI-320-GFPi is nearly unresponsive to the inducer IPTG, suggesting that it interferes with lactose binding.

We expected LacI to be tolerant of insertions in the hinge region between the N- and C-terminal core domains [27], [30], but the repression activity of LacI-158-GFPi is down three orders of magnitude from LacI-312-GFPi. Residues 151–158 of LacI form a mutationally tolerant hinge that connects the N-terminal core domain to the C-terminal core domain that is in close spatial proximity to the loop that contains residue 312. It is likely that the 237 codon GFP insertion in this region is more detrimental to protein function than the 31 codon epitope insertion [30] because it is much larger.

#### Dimerization interface

The functional unit of LacI is a tetramer comprised of a dimer of dimers. Proteins that assemble into dimers, tetramers, polymers, etc. pose a greater challenge when identifying sites that can tolerate GFP insertion. Four principle clusters of amino acids are involved in dimerization (159–163, 221–226, 251–259, 280–285) [27], [40]. We were surprised to find that an insertion near one of these sites, LacI-221-GFPi, retains some repression activity. The crystal structure of LacI reveals that amino acid 221 is at the end of a short linker region adjacent to the second alpha helix of the C-terminal core domain. It is possible that the long linker connecting LacI to GFP encoded by TAGIT may be sufficiently flexible to allow LacI dimerization.

#### Tetramerization domain

In LacI-351-GFPi, GFP is inserted nine amino acids from the C-terminus (Figure 4B and 4C) and is almost equivalent to the C-terminal GFP fusion protein typically used to localize DNA molecules in living cells. As expected, LacI-351-GFPi can repress the lactose operon, but it was less active than LacI-312-GFPi. Hence, the optimal site for GFP insertion is not at the N- or C-terminus and would therefore have been very difficult to identify using conventional GFP tagging methods.

### Potential Utility of Repression Defective LacI-GFPi Mutants

LacI-GFP fusions are commonly used to track the movement of plasmids or chromosome loci into which arrays of lactose operators have been integrated. Tracking the movement of chromosomes in growing cells using this method poses challenges because *lacI-gfp* can cause growth defects when expressed in cells that contain a chromosomal *lacO* array [31]. We found this to be the case for GFP insertions at amino acids 158, 283, 288, 312, 315, and 351 of LacI, and we found that filamentation was exacerbated by increased growth temperature (from room temperature to 30°C). We identified several LacI-GFPi proteins that allieviate these problems. GFP insertion proteins at amino acid 133, 143, 191, and 240 of LacI were unable to repress the lactose operon, but nevertheless retained sufficient DNA binding activity to localize as *lacO* array-associated foci (Figure 3, Figure S1).

## Materials and Methods

### Strains, Reagents, and Recombinant DNA Techniques

The following *E. coli* strains were used in this study: CSH140 [42], IL05 [31], DH5α [43], Top10 (Invitrogen) and KJ622 [44]. Restriction enzymes were purchased from New England Biolabs. Tn5 transposase was a gift from Dr. William Reznikoff (University of Wisconsin). DNA digestion and ligation reactions and transformations of *E. coli* were performed according to standard protocols [45]. Cultures were grown in Luria broth (LB) or M63 supplemented with 0.2% glucose or 1 mm IPTG as appropriate. When required, antibiotics were used at the following concentration: kanamycin (50 µg/ml), ampicillin (100 µg/ml).

### Construction of pTAGIT-1 and pTAGIT-2

Plasmid pTAGIT-2 was constructed in the following manner. Plasmid pMDS12 [46] was digested with the restriction enzymes BamHI and SpeI to isolate the fragment corresponding to the superbright *gfp* gene [47]. This fragment was gel purified and then ligated to a BamHI and SphI digested pUC19 vector [48] to yield pEB49. Next, we introduced the *kan* gene with its native promoter from plasmid pEB9. Plasmid pEB9 was constructed by amplifying the *kan* gene from pDG364 [49] by PCR using primers EB15 and EB16, which create a fragment containing the *kan* gene flanked by *loxP* sites. This fragment was digested with the restriction enzymes BamHI and SpeI and ligated to pMDS73 [46] that had also been digested with BamHI and SpeI to give pEB9. The *kan loxP* fragment was amplified using PCR from pEB9 using primers EB106 and EB128. This fragment was digested with SpeI and NotI, gel purified, and then ligated to pEB49 that had been digested with SpeI and NotI to give pEB118. The *loxP 'lacZ* fragment was amplified by PCR from pEB9 using primers EB105 and EB127. This fragment was cloned into pCR3.2-topo blunt (Invitrogen) and subsequently isolated by restriction digest with SpeI and AscI. We then constructed plasmid pEB123 by cloning the *loxP 'lacZ* fragment into SpeI and AscI digested pEB118. Plasmid pEB123 contains all the parts of TAGIT except for the ME (hyperactive mosaic end) that are recognized by the Tn5 transposase. To introduce the ME's we used primers JG10 and EB143, both of which contain the ME sequence, to amplify TAGIT from pEB123. This fragment was then poly A-tailed using Taq polymerase and ligated to SmaI digested pUC19 that had been poly T-tailed in the same manner to give plasmid pEB163. Plasmid pTAGIT-1 was constructed by digesting pEB163 with KpnI and SphI and ligating it to the conditional R6Kγ origin of replication [50], a modified origin from the R6K plasmid [51]. The R6Kγ origin was amplified by PCR with primers EB180 and EB181 from plasmid pRL27 [52]. To ensure that blue colonies were the result of in frame transpositions we used primers JG119 and JG120 to change the methionine codon near the 5′ end of *'lacZ* to a codon corresponding to alanine using the Quick-Change Site-Directed Mutagenesis protocol (Stratagene, La Jolla, CA).

### Construction of *lacI-gfpi* Library

The target plasmids, pEB363 and pEB364, were constructed by amplifying and cloning *lacI^q^* from pMUTIN-GFP+ [16] into the pSMART vector (Lucigen) using primers EB231 and EB232. The two target plasmids contain the *lacI^q^* insert in the opposite orientation. *In vitro* transposition was carried out using pTAGIT-2 and either pEB363 or pEB364 using Tn5 transposase. The transposition was transformed into XL-10 Gold competent cells (Stratagene) and plated on LB with kanamycin. The resulting transformants were pooled and plasmid DNA was isolated using QIAprep miniprep columns (Qiagen). The resulting plasmid DNA was transformed into the *pcnB* strain KJ622 and plated on LB with kanamycin and Xgal. Blue colonies were purified and plasmid DNA was prepared and transformed into Strataclone Solopack competent cells (Stratagene). This strain transiently expresses Cre recombinase and successfully excises the *kan* and *'lacZ* genes in approximately 80% of transformants. After Cre mediated excision, plasmid DNA corresponding to each of the blue colonies was prepped and sequenced using primers JG33 (downstream) and EB46 (upstream) to determine the position of *gfp* insertion in *lacI*.

### β-Galactosidase Assays

β-galactosidase activity was measured as Miller units in strain CSH140 transformed with each *lacI-gfpi* plasmid separately, pEB363, and pEB364. β-galactosidase activity was identical in CSH140 containing pEB363 or pEB364. Strains were grown overnight in LB ampicillin. Cultures of LB with ampicillin with or without IPTG (1 mM) were then inoculated with 20 µl of the overnight and grown to an OD_600_ of 0.4–0.6. Assays were then carried out as described [53]. The strain harboring *lacI-221-gfpi* was grown in M63 salts supplemented with glucose to ensure the retention of the F plasmid that contains the lactose operon. Optical densities were measured with a Beckman DU640 spectrophotometer. Repression activity was calculated as the ratio of the β-galactosidase activity of CSH140 to the β-galactosidase activity of CSH140 containing the appropriate plasmid grown in the appropriate media.

### Quantification of LacI-GFP Insertion Protein Levels by In-Gel Fluorescence

Protein accumulation of LacI-GFPi protein was measured by in-gel fluorescence [37]. The same strains used to measure β-galactosidase activity were grown in LB with ampicillin to an OD_600_ of 0.5. Approximately 1.0 OD_600_ of cells were pelleted by centrifugation and resuspended in 100 µl of SB buffer (140 mM Tris-HCl pH 8.8, 14% glycerol, 3.5 mM EDTA, 0.02% bromophenol blue, 0.05 M DTT, 4% SDS) Samples were analyzed by 12.5% SDS-PAGE and scanned using a Typhoon 9400 variable mode imager followed by coomassie staining. Quantification was performed using ImageQuant 5.2. Relative protein levels were reported as a ratio of the fluorescence of each sample to the fluorescence of LacI-99-GFPi, which had the lowest protein accumulation of all the samples.

### Microscopy

Strains were prepared by transforming strain IL05 with each *lacI-gfpi* plasmid. Transformants were grown on LB with ampicillin and IPTG. All microscopy was performed using LB agar pads without antibiotics as described previously [13] at 30°C or at room temperature. Images were acquired using an Applied Precision Spectris microscope and deconvolved using softWoRx version 3.3.6 software (Applied Precision). Figures were assembled with Photoshop CS.

### 3D Cartoon Model

The three-dimensional structure of the lactose repressor (PDB ID: 1LBI and PDB ID: 1LBG; [40] was manipulated using Visual Molecular Dynamics (VMD ver. 1.8.6).

## Supporting Information

Figure S1 Binding of LacI-GFPi proteins (green) to *lacO* arrays near *ter* in growing *E. coli* cells stained with FM 4–64 (red). Numbers correspond to the codon after which TAGIT was inserted. The complete set of unique insertions is shown here.
(1.31 MB TIF)
