# Supplementary figures and images for: Transposon Assisted Gene Insertion Technology (TAGIT): A Tool for Generating Fluorescent Fusion Proteins
Source: PLoS One. 2010 Jan 15;5(1):e8731. doi: 10.1371/journal.pone.0008731 (PMC2806921; doi:10.1371/journal.pone.0008731)

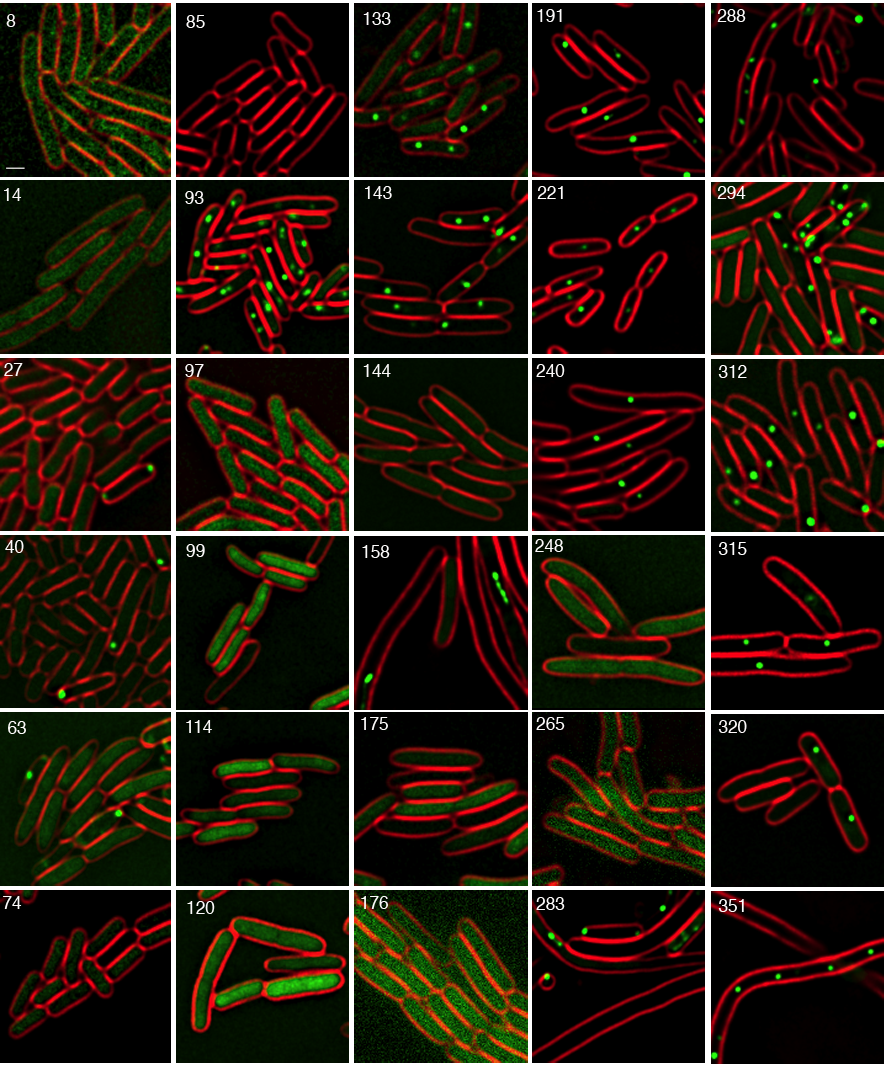

Supplement: Figure S1 — Binding of LacI-GFPi proteins (green) to lacO arrays near ter in growing E. coli cells stained with FM 4–64 (red). Numbers correspond to the codon after which TAGIT was inserted. The complete set of unique insertions is shown here. (1.31 MB TIF) [file pone.0008731.s001.tif]
